# Biosensing for the Environment and Defence: Aqueous Uranyl Detection Using Bacterial Surface Layer Proteins

**DOI:** 10.3390/s100504739

**Published:** 2010-05-10

**Authors:** David J.R. Conroy, Paul A. Millner, Douglas I. Stewart, Katrin Pollmann

**Affiliations:** 1 Biosensors and Biocatalysis Group, Institute of Membranes and Systems Biology, University of Leeds, Leeds, LS2 9JT, UK; E-Mail: p.a.millner@leeds.ac.uk; 2 School of Civil Engineering, University of Leeds, Leeds LS2 9JT, UK; E-Mail: d.i.stewart@leeds.ac.uk; 3 Institute of Radiochemistry, Forschungszentrum Dresden, Rossendorf, Germany; E-Mail: k.pollmann@fzd.de

**Keywords:** S-layer, surface layer, protein biosensor, uranium, uranyl, metal ion, sequestering, impedance spectroscopy

## Abstract

The fabrication of novel uranyl (UO_2_^2+^) binding protein based sensors is reported. The new biosensor responds to picomolar levels of aqueous uranyl ions within minutes using *Lysinibacillus sphaericus* JG-A12 S-layer protein tethered to gold electrodes. In comparison to traditional self assembled monolayer based biosensors the porous bioconjugated layer gave greater stability, longer electrode life span and a denser protein layer. Biosensors responded specifically to UO_2_^2+^ ions and showed minor interference from Ni^2+^, Cs^+^, Cd^2+^ and Co^2+^. Chemical modification of JG-A12 protein phosphate and carboxyl groups prevented UO_2_^2+^ binding, showing that both moieties are involved in the recognition to UO_2_^2+^.

## 1. Introduction

### 1.1. Toxicology of Uranium

Toxicologically, the uranyl ion is hazardous due to rapid adsorption through the gastrointestinal tract. In the bloodstream most uranyl is carried as soluble bicarbonate while the remainder is bound to plasma proteins. Whilst typically 60% is excreted within 24 h approximately 25% has been shown to undergo incorporation to bone [1]. Historic experiments in human test participants showed systemic exposure above levels of 0.1 mg/kg body weight results in acute renal tubular damage that can be fatal. There are currently no diagnostic tests available and no proven methods for reducing the chronic effects of uranyl exposure [2] which is why the application of biosensing for early detection of contaminated aqueous systems would be advantageous.

### 1.2. Environmental Effects of Uranium

In the UK uranium based hazards arise from fuels, materials and wastes produced from United Kingdom Atomic Energy Authority (UKAEA) and British Nuclear Fuels plc (BNFL) dating back to the 1940s and 1960s as well as Magnox power stations from the 1950s to 1970s. High level radioactive waste is buried with the intention of radioactive decay reducing activity over significant periods of time. Even minor leakage and radionuclide migration through container vessels can result in disastrous environmental ramifications [3]. Sellafield Ltd (previously British Nuclear Fuels) is a U.K nuclear processing site, and one of the few UK sites that publicly discloses information on soil analysis and allows a realistic model of the environmental contamination to be made. By-products from the nuclear fission process are the radionuclides U-238, Tc-99, Sr-90 and Cs-137. Of the five oxidation states of uranium only +4 and +6 are stable for practical considerations. The +6 species forms the water soluble uranyl (UO_2_^2+^) ion and is the most commonly encountered form.

### 1.3. Current Sensing Technologies

Three remediation strategies currently used for ground water remediation; (i) natural attenuation systems use reactive elemental reducing agents that induce abiotic degradation of substances (ii) wetland and mine effluents sorbtion systems and (iii) permeable reactive barriers (PRB) for ground water remediation that act as large scale sorption or reductive-precipitation barriers that sequester contaminants *in situ* over extended time scales. Economical and political restrictions often resist implementation of these methods. In such situations as these, the application of biosensing technologies is the most practical solution to continually monitor a target site where a complete remediation strategy is not possible. Current metal ion detection systems are limited, often with poor specificity and are limited to laboratory analyses. Chemical modification of surfaces to create chelator coatings can work as sorption barriers but they tend to lack specificity for analytes [4]. Alternative mass based systems use microcantilevers that monitor concentration changes of metal ions present as a function of frequency dampening have been developed [5] but lack specificity; if a conformational change in the binding protein occurs as a result of analyte binding [6] monitoring such a mechanism is relatively easy. If binding induces structural changes in a protein then even fM concentrations of the analyte can result in large mass and interface changes that are readily measurable [7]. For example, at a magnitude of size smaller, oligonucleotide sequences generated by PCR can be identified using enzymes and chronocoulometry [8]. The current limitation for these approaches is simply that too few analyte specific binding proteins have been discovered. Similarly enzyme based systems that use metal ions to enhance or inhibit a reaction in a quantifiable analyte specific manner has been shown [9] but are limited in number. Most proteins do not undergo a conformational change on binding and so analyte binding cannot simply be monitored by a change in interface mass.

### 1.4. Bacillus Sphaericus S-layer Proteins

While a few bacterial strains have been identified e.g *Pseudomonasstutzeri, Neurospora sitophila, Streptomycesalbus* and *Streptomyces viridochromogenes* [10] that are tolerant to, and able to sequester uranyl ions, the specific mechanisms and bindings sites are poorly understood. Bacteria regulate their response to specific metals by a number of mechanisms. Membrane pumps use an active potential to translocate ions from the cell by pumping out metal ions from the bacteria and maintain ion concentrations below toxic levels. However, many bacterial species have evolved specific proteins, externally or internally, that that bind and sequester metal ions to minimise uptake [11]. *Bacillus sphaericus* strain JG-A12 has evolved naturally under chronic exposure to uranium mining waste within piles near the town of Johanngeorgenstadt (Saxony, Germany [12]). This strain shows an intrinsic tolerance to the radioactive compound [13]. Compared to similar strains, JG-A12 was reported to bind uranyl ions with higher specificity. Early reports [20] claimed JG-A12 showed specificity only for UO_2_^2+^ making it an ideal metal receptor. However subsequent work [14] monitored the interaction of this strain with 19 heavy metals (Al, Ba, Cd, Co, Cr, Cs, Cu, Fe, Ga, Mn, Ni, Rb, Si, Sn, Sr, Ti, U, and Zn). While failing to bind a number of divalent ions that similar strains could bind, JG-A12 bound Cu, Pb, Al, and Cd to a small extent as well as UO_2_^2+^ [15]. Thus, while not offering complete specificity to uranium it binds to a fewer number of interfering cations than related species and has a significantly higher affinity for UO_2_^2+^.

### 1.5. Electrochemical Biosensors

Electrochemical biosensors typically employ a binding protein of some sort as the recognition element and are of increasing interest due their simplicity of operation and low cost of fabrication. They also show potential for near real-time detection and excellent specificity [16]. Current examples include, but are not limited to, medical diagnostics and serodiagnosis [17] tumour marker analysis [18], early identification of tissue damage [19] and cardiac marker analysis. However, most of these biosensors are designed to quantify larger analytes such as proteins. For much smaller analytes such as metal ions, several classes of proteins exist that chelate, transport or remove them, either as a natural function or to avoid cytotoxicity. Metallohistins are a recent class of histidine rich metal binding proteins found in the plant *Alnus glutinosa* [20]. Phyto-chelatins are metal chelating peptides important for heavy metal regulation in certain plants, fungi and bacteria containing the binding sequence (γ-Glu-Cys)n-Gly [20] and some have been found to bind Cu and Zn for storage in both eukaryotes and prokaryotes [21]. A range of bacteria and some eukaryotic algae contain a highly ordered array of surface layer proteins (SLP) or glycoproteins creating a porous outer shell. The biological roles can be specific to the organism but can include cell adhesion, protection from predation, virulence factor, antigenic properties, anchoring sites for exoenzymes or porin function [22], typically the layer lattice is 5–10 nm deep with pores of 2–6 nm diameter. Upon isolation, purification and re-suspension these form ordered 2 dimensional arrays on lipid or solid supports with crystalline arrays of oblique (p1, p2), tetragonal (p4), or hexagonal (p3, p6) symmetry with between 1 and 6 protein subunits [23]. The anchoring mechanisms of these proteins to cells vary, but include interactions with hydrophobic mycolic acid tails, template support layers on the cell surface or orientated nanogrooves for protein assembly and ordering [24].

#### 1.5.1. Electrochemical Impedance Spectroscopy

Electrochemical impedance spectroscopy (EIS) is a method of interrogating surfaces and interfaces as a function of current dissipation with frequency. Specifically to biosensing, the changes in resistance and capacitance in response to an analyte-interface interaction can be observed. Impedance is the ratio of current change to a incremental applied voltage and has emerged as a powerful technique for monitoring interfacial changes at a solid-liquid or liquid-liquid interface for a number of biosensing mechanisms including membrane-analyte interactions [25], ion channels [26], interfacial capacitance changes [27] and antibody/antigen interactions [28].

Models of EIS idealise an electrode interface as a series of electronic circuit components which are used to model current dissipation with frequency. Models of increasing complexity use resistors and capacitors in series and parallel to represent the resistance and capacitance changes at an electrode interface due to mass transport phenomena or reaction transfer kinetics of species at the interface. Bulk impedance (Z) can be expressed as a complex function represented as the sum of the real Z’(ω) and imaginary −Z”(ω). These are the resistance and capacitance components respectively and is typically represented as a Nyquist plot which shows the imaginary −Z” part on the Y axis and the real Z’ part on the X axis. Interpreting the Nyquist plot using a representative equivalent circuit model shows changes in impedance from interfacial phenomena such as analyte binding as a function of solution resistance, interfacial resistance and layer capacitance [29].

## 2. Results and Discussion

### 2.1. Surface Preparation

Two alternative protein tethering mechanisms of SLP were performed. However, it is essential a clean uniform base gold layer is prepared for repeatable layer by layer depositions and subsequent biosensor construction. Thus a number of surface cleaning routines were performed. Ozone and chemical etchants, produced the cleanest electrodes compared to surfactant and solvent washes, but caused significant surface damage with repeated use. The effects on surface roughness and area change these methods induced were calculated using the Cottrell equation which relates the current decay of a potential ramped electrode in solution with an electro active species [30]. Up to 2 min in piranha solution (a highly exothermic and corrosive mixture of 7:3 (v/v) H_2_SO_4_ and H_2_O_2_) yielded clean electrodes with minimal surface damage, whilst 15 min piranha washes created surface roughness factors showing up to a tenfold increase in surface area. As a result, a 2 min piranha wash followed by a rinse with methanol and isopropyl gave the optimal gold layer.

### 2.2. Analysis of Sensor Fabrication

Two methods of tethering the SLP were optimised; a mixed self assembled monolayer (mSAM) was compared to a porous membrane bioconjugation method. Incorporation of surface layer protein (SLP) was optimised using increasing ratios of 16-mercaptohexadecanoic acid (MHDA) to 1,2-dipalmitoyl-sn-glycero-3-phosphoethanolamine-N-(cap biotinyl) (biotin-caproyl-DPPE) in the mSAM. An increased ratio of biotin-caproyl-DPPE showed an increase in the binding sites of the docking protein Neutravidin and thus the binding density of biotinylated SLP. However beyond a 50% (mol/mol) ratio a breakdown of the mSAM was seen and caused the formation of independent stable domains of the mSAM components [27]. A 20% (mol/mol) ratio of biotin-caproyl-DPPE to MHDA) was determined to be the optimal amount for mSAM stability. Successful Neutravidin adsorbtion onto the mSAM was monitored by quartz crystal microbalance (QCM). System instability occurred upon SLP tethering to a biotin tagged mSAM. The possibility that the S-layer protein was directly inserting into the mSAM was unlikely due to the JG-A12 SLP isoelectric point theoretically calculated as pH 5. At pH 7 both protein and mSAM are negatively charged. Extensive X-ray reflectivity studies on similar SLPs from bacterial strains CCM2177 and E38-66 on DPPE (a cationic lipid that binds to negative protein regions) did show protein adsorption onto the lipid head groups resulting in some intercalation at least up to the phosphate moieties and probably further [31]. It is unlikely that the SLP was disrupting the mSAM and the instability was most likely due to the viscoelastic nature of the linkers introducing dispersion affects. Addition and tethering of biotinylated-SLP could not be achieved reproducibly and thus a bioconjugation approach was chosen for the optimised biosensor.

**Figure 1.**
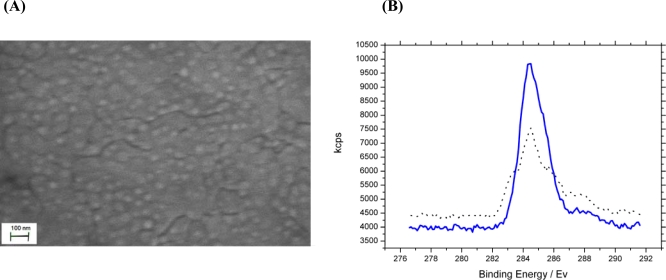
(A) SEM image of organic SLP biosensor layer bound to a gold working electrode. Dense protein layer covalently bound with boundaries between protein domains. (B) XPS analysis of surface composition of top 5nm of bound protein layer before ( 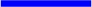) and after ( 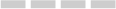) chemical modification of phosphate and carboxylate groups. The data show a 30.2% carbon C 1s to gold 4f peak ratio increase confirming successful modification of analyte binding sites.

Successful layer by layer deposition of the bioconjugated tethering layer was confirmed by EIS. Nyquist analysis showed that while 4-aminothiophenol (4-ATP) binds within the first hour, stabilisation and ordering of the molecular layer to an ordered SAM occurred beyond 4 hrs, thus a minimum of 4 hrs incubation was required. To covalently attach the cross linker 4-(N-maleimidomethyl)cyclohexane-1-carboxylic acid 3-sulfo-N-hydroxysuccinimide ester (sulfo–SMCC) which reacts with the amine moiety of the 4-ATP SAM coated electrodes, a further incubation in 5 mM sulfo-SMCC PBS pH 7 solution at least 1 h was performed. The free maleimide groups present bound to free cysteine sulfhydryl groups on the SLP creating a covalently tethered protein layer. SEM analysis of the electrode surface after deposition of the SLP (Figure 1A) produced a very uniform image. This is because a dense protein layer was successfully covalently linked to the bioconjugation layer separated by flat regions that acted as boundaries between protein domains. Extended imaging resulted in charging burns that caused permanent damage to the biolayer but confirmed the successfully covalently bonded protein layer. Atomic force microscopy allowed analysis of the sensor surface physical properties. The 4-aminothiophenol layer created a planar layer of linkers separated by their own electrostatic charge from the aromatic ring. These were linked to the linear sulfo-SMCC groups creating a total linker approximately 1.5 nm in length. However rather than acting as a solid anchor to tether the protein the linker sulfo-SMCC appears to have acted as a flexible spring-like linker shown by the lateral and compressive deviation in tip tapping mode analysis. As a result the proteins deviated from the microscope probe resulting in trough formation parallel to the scanning direction. This suggests an almost fluid like interface rather than solid linkers. The total bioconjugated linker layer at preferred orientation extends approximately 1.5 nm from the gold surface. Also, due to the repulsive nature of the probe in regards to the protein’s negative charge above its isoelectric point some degree of protein deviation was expected. The soft-fluid interfacial data supports a model in which the surface acts as a porous membrane interface and also explains the need for electrode equilibration with each batch of electrodes which took at least 30 min upon immersion in electrolyte. X-ray photoelectron spectroscopy (XPS) analysis (Figure 1B) demonstrated successful deposition of each incubation layer and also allowed phosphate and carboxylate modification to be followed. Protein attachment was observed as a significant gold Au 4f to carbon C 1s peak ratio. A significant carbon increase, on average 30.2% carbon C 1s to gold 4f peak ratio increase on chemically modified SLP biosensors, supports the idea that phosphate binding groups were, as intended, successfully modified by acylation, as were carboxylate binding groups by tris(hydroxymethyl)aminomethane modification.

### 2.3. Binding of UO_2_^2+^ to the SLP Biosensor

Binding of a reversible protein layer under an electric field adds an additional capacitive component [32]. Covalently linking such a layer creates a more stable interrogatable interface. In addition to protein capacitance, the capacitance between electrode and an ion in solution and the electrode is modelled as series of capacitors (Equation (1)):

(1) $$\frac{1}{C}=\frac{1}{C_{\text{mod}}}+\frac{1}{C_{\mathrm{dl}}}$$

Where C_mod_ is the modifier layer of absorbed species and C_dl_ the capacitance of the natural double layer occurring at a liquid-electrode interface as modelled by Gouy Chapman–Stern theory [29]. Simple mSAM based systems can often be modelled by use of a parallel capacitor and resistor in series to a second resistor (the Randles circuit). Comparison of modified and unmodified layers can be used to show the distribution of defects, pinholes, the effect of linked redox probes and the kinetics and mechanism of the monolayer formation process [33]. However, increasing model complexity by adding increasing components to accurately model organic-metallic interfaces is not usually justified because many of the imperfections of natural surfaces and roughness of electrode substrates [34]. In addition, lateral inhomogeneities between mSAM component molecules means multiple equivalent circuits often fit impedance data without accurately modelling the system [35]. Binding nanomolar levels of atoms to the Stern layer will cause a small disruption to the outer Helmholtz plane of the Guoy-Chapman model. Because these processes occurred on the nanometer scale they are difficult to detect over other dominating processes. Use of buffer concentrations magnitudes greater than the analyte monitored allowed agreement between Gouy–Chapman–Stern (GCS) model and experimental results observed in dilute solutions near the point of zero charge [36], minimising the changes in C_dl_ in response to analyte addition. Binding of analytes to the absorbed molecular layers thus caused an increase in modified layer capacitance (extending the closest distance of approach of a molecule, increasing the resistive component of the inner Helmholtz Plane (IHP) and a decrease in double layer capacitance due to a compression in the double layer. Equation (2) shows the dominant of these opposing processes will determine if binding causes an increase or decrease in C_dl_, the double layer capacitance, C_protein_, the additional capacitance component from the protein layer, and C_analyte_, the capacitance from addition of a charge species binding at the interface.

(2) $$\frac{1}{C_{\mathrm{dl}}}=\frac{1}{C_{\mathrm{protein}}}+\frac{1}{C_{\mathrm{analyte}}}$$

**Figure 2.**
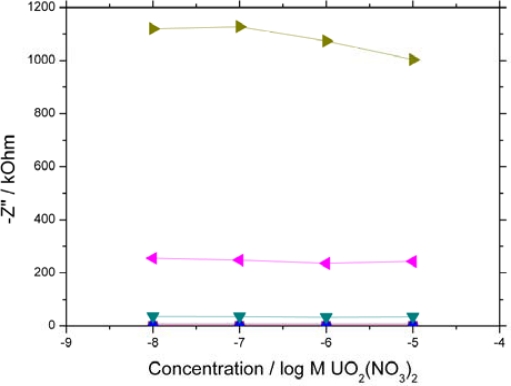
Dependence of imaginary component of impedance on uranyl ion concentration. The JG-A12 SLP based sensors were exposed to UO_2_(NO_3_)_2_ and stirred continuously for 15 mins before a 30 min equilibration period. EIS scans were performed in 10 mM PBS at 0 V *vs.* Ag/AgCl at a perturbation of 10 mV. The signals at (▪ 10 kHz, 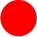 1 kHz, 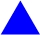 100 Hz, 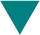 10 Hz, 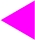 1 Hz, 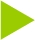 0.1 Hz) are shown. 10 kHz – 100 Hz all overlay each other close to zero due to the system exhibiting high resistive and low capacitive behaviour at high frequency.

Analyte binding caused a disruption in the interfacial double layer by disrupting the hydrated salt layer in the outer Helholtz plane (OHP). By plotting the Nyquist data as a function of concentration response to different frequencies (Figure 2) a mass transport response is observed at low frequencies.

Figure 2 shows a significant decrease in the imaginary component of impedance at lower frequencies in response to increasing analyte concentration. Poration of the interface between proteins allowed charged analyte to be delivered to and from the interface. Successful binding to the protein layer with increasing analyte concentration increases the charge density across the interface, increasing the layer capacitance and decreasing the imaginary impedance component. As a result more information can be obtained about interface mechanisms at low frequencies as mass transport to the interface is the limiting step compared to the electron transfer kinetics at high frequencies.

**Figure 3.**
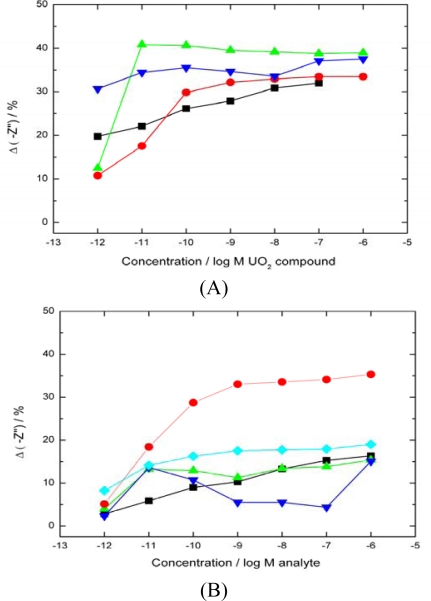
Real time capacitance response to analytes. Sensors were exposed to increasing concentrations of analyte and stirred continuously for 15 mins before a 30 min equilibration period. EIS scans were performed in 10 mM PBS at 0 V *vs.* Ag/AgCl at a perturbation of 10 mV at 0.1 Hz. (A)–Response of biosensor to different uranyl compound response (▪ Uranyl nitrate on 6 hr old electrode, 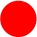 uranyl nitrate response from a 7 days old electrode, 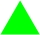 natural uranyl nitrate response 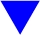 uranyl acetate response). The data shows no differentiation between uranyl compounds as all are able to bind with the UO_2_^2+^ in the +6 oxidation state. (B)–Response of biosensor to a range of interfering divalent cations (▪ nickel nitrate, 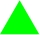 caesium sulphate, 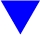 cadmium nitrate, 
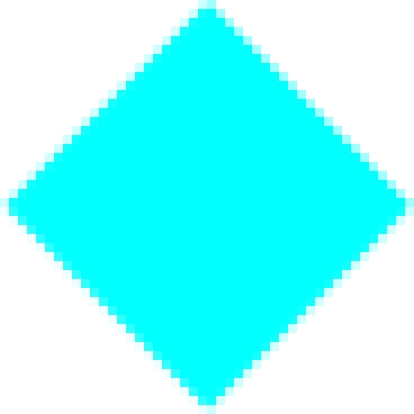
 cobalt chloride, 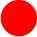 average uranyl response).

Data from low frequency scans with a response to a range of UO_2_^2+^ compounds is shown in (Figure 3A). EIS data for aqueous binding systems is almost always sigmoidal be it antibody, protein or chemo-receptor based systems. This is a logical consequence of the relationship between receptor-ligand complex and ligand concentration in contrast to linear responses that are often observed with amperometric systems in which a direct analyte to product current is generated. The result of analyte binding to the protein layer caused an increasing charge build up at the interface building to saturation. Experimental repeats all lay within an average curve with error bars of ±2 standard deviations. Within the centre region 10^−11^ M to 10^−7^ M, between biosensor lower limit and saturation point respectively a linear response is observed. The sensor is still functional above and below this range but yields a less accurate response. However, if a sample gives a response outside the linear range it could either be diluted or concentrated to lie within the linear range on the calibration plot.

**Figure 4.**
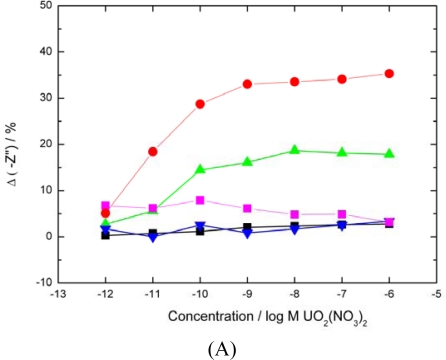

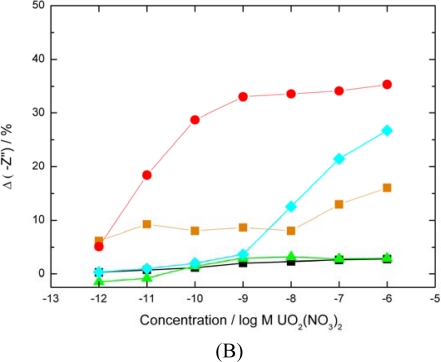
Effect of using non-specific proteins as the sensing agent. (A) Biosensors were constructed and the response to UO_2_^2+^ monitored ( 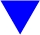 Casein sensor response, 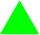 BSA sensor response, 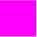 BSA sensor response with carboxylates blocked, 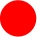 average uranyl response of SLP biosensor for comparison). The percentage decrease in –Z” was calculated as previously. Sequential analyte injections were performed over a 6 hours period. (▪ A control sensor with no analyte added showed only a 2% drift in base signal over the same period). (B) Modified SLP protein response to UO_2_^2+^ ( 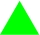 Both carboxylates and phosphates moieties blocked 
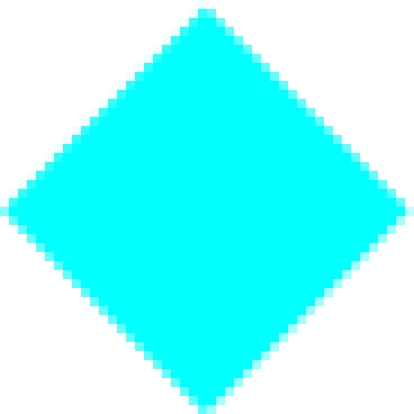
 carboxylates only blocked, 
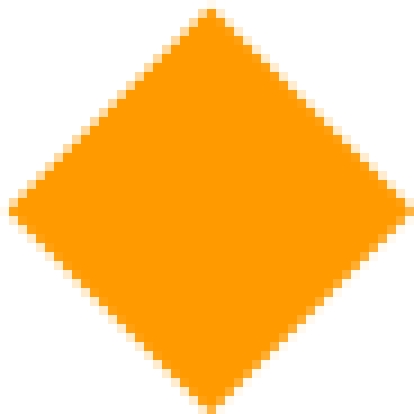
 phosphates only blocked, ▪ base signal drift over a 6 h period, 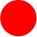 average uranyl response of SLP biosensor for comparison).

Binding of uranyl ions to the protein layer resulted in a large decrease in the imaginary impedance component, significantly more than other interfering divalent cations (Figure 3B). Significant charge build up at the interface from analyte binding appeared to compress the molecular double layer showing the greater selectivity of the JG-A12 SLP to UO_2_^2+^ than other analytes (Ni, Cs, Cd, Co on Figure 3B). Analytes for which JG-A12 SLP had a lower affinity caused a smaller decrease in imaginary impedance, typically around 10–20%. To confirm this apparent selectivity for UO_2_^2+^ was due to binding by the JG-A12 SLP a number of controls were performed using alternative protein layers and binding site modification (Figure 4).

Sequential uranyl aliquots were added to a bare electrode in buffer in comparison showed a −Z” response 3 orders of magnitude lower showing that which there is some double layer capacitive component from unbound uranyl ion-gold interaction it is significantly lower than the main signal. The signal stability of a bare electrode in only buffer was monitored over 6 hrs and was stable within 2% of the base signal during this time. Sensors constructed of other proteins that lacked the uranyl binding specificity of JG-A12 SLP showed a lower binding response (Figure 4A). The phosphoprotein casein was used to further elucidate if the sequestering ability of JG-A12 SLP originated via a monodentate mechanism using phosphate groups or a bidentate mechanism involving both phosphate and carboxylate groups. This is because the JG-A12 SLP is similarly a highly phosphorylated protein. Bovine serum albumin (BSA), a relatively stable and inhert protein often used to block non specific analyte binding was similarly used as a control. There was virtually no response from the casein sensors which showed that in spite of a high degree of phosphorylation, the protein did not manage to bind a significant amount of uranyl. This supports the idea that the JG-A12 SLP was responsible for the specific UO_2_^2+^ binding. BSA gave an intermediate response due to the non-specific electrostatic binding of UO_2_^2+^ to the proteins negative surface charge. These two alternate protein sensors support the specific binding of JG-A12 SLP to uranyl in a bidentate manner. Figure 4b shows individual and combined functional group blocking on a functioning SLP biosensor. While there is some limited response when only 1 binding site is chemically blocked suggesting some monodentate binding, the complete binding inhibition by blocking both functional groups supports a dominating bidentate mechanism. Experiments also showed that uranyl binding was reversible as would be predicted since the interaction mechanism is non-covalent. Previously uranyl saturated biosensors that had brief buffer washes showed complete removal of uranyl ions which strongly implies rapid on and off rates for the UO_2_^2+^ binding to the SLP.

## 3. Materials and Methods

### 3.1. Chemicals and Reagents

SLP from strain *Lysinibacillus sphaericus* JG-A12 was provided by Dr Katrin Pollmann, Institute of Radiochemistry, Forschungszentrum Dresden - Rossendorf, Germany. Transducers used were design P3 [28] comprising a 1 mm diameter gold working electrode fabricated on a SiO_2_ coated Si wafer over a Ti adhesion layer. These were sourced from the Tyndall institute, Cork. 4-aminothiphenol (4-ATP), 4-(N-maleimidomethyl)cyclohexane-1-carboxylic acid 3-sulfo-N-hydroxysuccinimide ester sodium salt (sulfo-SMCC), 16-mercaptohexadecanoic acid (MHDA) and biotin-N-Hydroxysulfosuccinimide (biotin-NHS) were obtained from Sigma-Aldrich. Biotin-caproyl-DPPE was obtained from Avanti Polar lipids whilst Neutravidin was acquired from Pierce. All other solvents and buffers unless stated obtained from Sigma-Aldrich.

### 3.2. Electrochemical Setup

EIS was performed on PGSTAT100 FRA and microAutolabIII/FRA2 systems. Experiments were performed with a gold P3 working electrode, an Ag/AgCl reference electrode and a solid platinum counter electrode, in a classic 3 electrode system. Phosphate buffered saline solution at pH 7.0 comprising 140 mM NaCl, 2.7 mM KCl, 0.1 mM Na_2_HPO_4_ and 1.8 mM KH_2_PO_4_ was used as the supporting electrolyte. A range of cleaning methods was used for preparation of the gold electrodes and then the amperometric response monitored by cylclic voltammetry scans in 5 mM potassium ferricyanide as the redox probe. Cleaning protocols tested were ethanol wash and sonication, 15 min UV irradiation followed by ethanol wash, ozone treatment followed by an ethanol wash, 2 mins in a piranha solution (7:3 v/v sulphuric : hydrogen peroxide), 5 mins in a 7:3 (v/v) piranha solution, 15 mins in a 1:1 piranha solution. Piranha solution, gives an aggressive treatment that erodes metal with excessive use, and thus reduced electrode life span with extended use, but also offers the most powerful organic removal from electrode surfaces. The optimum method (see Results) was 2 min clean in a 7:3 (v/v) piranha solution.

### 3.3. SLP Tethering Mechanisms

**Figure 5.**
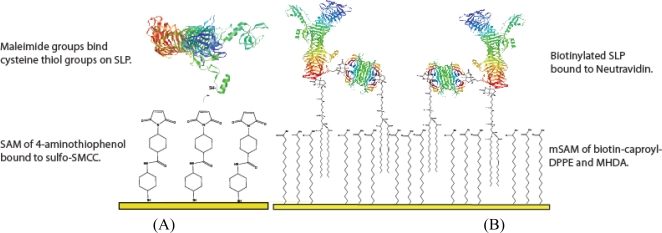
Schematic showing the two alternate tethering methods for SLP incorporation on to gold surfaces. (A) mSAM incorporation of SLP by MHDA/biotin-caproyl-DPPE mSAM, deposited with a Neutravidin layer that binds to pre-biotinylated SLP. (B) Porous membrane model with molecular linkers of 1.5 nm length binding SLP through a stable permeable membrane as maleimide groups covalently bind to thiols on protein cysteine residues.

Two alternative tethering mechaisms of the SLP were performed, a schematic representation shown in (Figure 5A), Neutravidn-biotin mSAm tethering and (Figure 5B), the gold bioconjugation method.

#### 3.3.1. Biotin-Neutravidin mSAM preparation

For mSAM preparation initially a 10:1 (M/M) biotin-caproyl-DPPE to MHDA ratio was used. For this 44 μL of 10 mg/mL MHDA in ChCl_3_ was added to 10 ml EtOH to form a 0.5 mM/500 μM stock solution. To this 52.5 μL of biotin-caproyl-DPPE was added from a 10 mM stock in CHCl_3_ to create a total working concentration of 50 μM biotin-caproyl-DPPE and a 10:1 molar ratio of MHDA : biotin-caproyl-DPPE. Electrodes were incubated overnight to prepare the mSAM. Biotinylated SLP was bound to a Netravidin protein layer that was preassembled on a MHDA:DPPE mSAM [27]. In the present work n-hydroxysuccinimide activated carboxy biotin was used to biotinylate the SLP. The protein was dialysed for 24 hrs against PBS to remove interferants. Biotin/S-layer protein ratios tested were 1000, 100 and 10 to 1. A 10:1 ratio with a 30 min incubation time was found to be optimal for binding. Bound and unbound complexes purified by a PD-10 Desalting column (Sephadex G-25).

##### 3.3.1.1. mSAM stability measurements at varying MHDA : biotin-caproyl-DPPE

Varying ratios of biotin-caproyl-DPPE : MHDA were prepared in 10 ml ethanol to be absorbed on gold P3 electrodes. The mSAM was interrogated over a frequency range 250 kHz to 0.25 Hz. Fifty data points were measured to monitor self assembly. Readings were taken immediately upon electrode immersion into the component solution to monitor adsorption, assembly or stability as a function of capacitance.

#### 3.3.2. Bioconjugation layer preparation

Cleaned electrodes were incubated in 10 mM 4-ATP in ethanol solution for 4 hours. Hourly Nyquist scans showed that while a significant amount of 4-ATP bound within the first hr, stabilisation and molecular ordering occurred sometime after 4 hours. To attach sulfo–SMCC the electrodes were then incubated in a 5 mM sulfo-SMCC in PBS pH 7.0 for a minimum of 1 hour. Sulfo-SMCC binds to amine groups of the 4-ATP monolayer; its maleimide groups are then free to bind cysteine thiol groups on the SLP. Once the supporting layer had been prepared, the electrodes were further incubated for a minimum of 1 hr in 1 mg/ml protein and stored in 10 mM PBS at room temperature. Electrodes were interrogated after each deposition step to confirm successful layer deposition.

### 3.4. Blocking S-the SLP Chelating Sites

**Figure 6.**
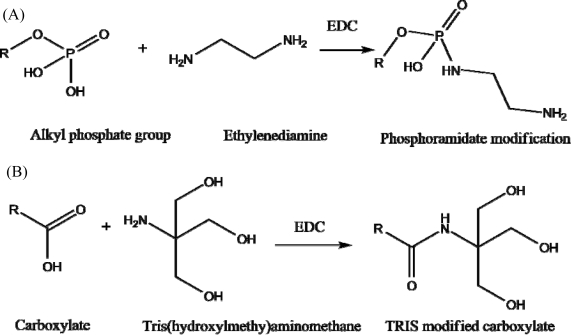
Schematic of the protocols used to modify proposed SLP analyte binding carboxylates and phosphates sites. (A) Phosphate modification by carbodiimide reaction in the presence of amine. (B) Modification of carboxylates with TRIS using carbodiimide mediated process.

The proposed binding mechanism of uranyl ions to JG-A12 SLP is through carboxyl and phosphate groups in a bidentate manner or via phosphate groups with monodentate orientation, the mechanism unique to JG-A12 SLP [14]. To confirm the impedance response change was due to the specificity of JG-A12 SLP to uranyl ions these sites were chemically modified (Figure 6). Carboxyl groups were blocked using acylation that created 3 terminal hydroxyls that introduced electrostatic and steric hindrance to analyte cations that attempted to bind. This was achieved by incubating an SLP bound electrode in 0.1 M MES pH 4.7 with 0.1 M TRIS with 10 mg/mL 1-ethyl-3-(3-dimethylaminopropyl) carbodimide for 4 hrs at room temperature. Phosphate blocking was achieved by phosphoramidate modification which added an amine to phosphate groups. This was performed by incubating at S-layer coated electrode in 5 mM ethylenediamine with 1-ethyl-3-(3-dimethylaminopropyl) carbodiimide under alkaline conditions (pH 7–10).

### 3.5. Surface and Construction Analysis

Atomic force microscopy was performed using a Nanoscope IV Pico scope force module. Scanning electron microscopy was performed on a Philips XL30 SEM. X-ray photoelectron spectroscopy was performed on a VG Escalab 250 XPS using a 500 μm spot size and 150 W power. QCM analysis was carried out using a Maxtek RQCM instrument using 5 MHz, 1 in. diameter AT cut crystals with Au coated surfaces. Real time deposition studies were obtained by setting up a flow through system using a 100 μL flow chamber and flow speed of 220 μL min.

## 4. Conclusions

It has been shown that by tethering protein layers to metal surfaces via bioconjugation it is possible to create a dense protein layer without denaturing the protein. Coating surfaces with proteins such as the JG-A12 SLP can create bio-functional surfaces; in the present report the SLP coated surface shows high specificity to UO_2_^2+^ ions. While mSAMs create stable environments for a number of enzymes and proteins, this was not the case for JG-A12 SLP and a more direct bioconjugation procedure proved more effective. Biosensors were shown to respond to sub-nM levels of aqueous uranyl with this response inhibited by chemical modification of proposed binding sites. The response from surfaces coated with control proteins supported our contention that the binding specificity was conferred by the JG-A12 SLP. Moreover chemical modification of carboxy and phosphate groups on the SLP abrogates uranyl recognition, indicating that the previous suggested binding mechanism [14] was correct. The current biosensor detection limit is 10^−12^ M. While a number of experiments were performed to 10^−15^ M these are currently difficult to reproduce and highly sensitive to system noise. A limit of 10^−12^ M and above has been reproducible across numerous protein and electrode batches. Our approach provides a new means of fabricating metal ion biosensors, and it is possible that SLP isolates from bacteria surviving in other metal polluted sites may provide the sensing components for fabrication of other metal ion biosensors.
